# The impact of foreign R&D on the innovation performance of China’s high-tech industry and its spatial spillover effect

**DOI:** 10.1371/journal.pone.0282626

**Published:** 2023-03-16

**Authors:** Xiaohong Han, Hua Feng

**Affiliations:** School of Economics and Management, Beijing Jiaotong University, Beijing, China; Gebze Teknik Universitesi, TURKEY

## Abstract

The inflow of foreign R&D has brought vigor and vitality to the development of the high-tech industry (HTI). Using the panel data of HTI in 23 provinces (autonomous provinces and municipalities) in China from 2007 to 2016, this paper firstly calculates the Moran index of HTI’s innovation performance (IP), and finds a spatial agglomeration effect. After rigorous testing, we determine the most suitable spatial metering model. Finally, the spatial effect is further decomposed into three kinds of effects: direct effect, indirect effect, and total effect. This paper studies the impact of foreign research and development (R&D) on IP of HTI and its spatial spillover effects. According to the research, foreign R&D has a significant role in promoting IP of HTI in China, and has specific spatial spillover effects. Significantly, foreign R&D has substantial positive spillover effects of space. When IP of HTI is measured by product innovation, there is no obvious space overflow. However, panel regression showed a significant positive effect. In terms of the influence on product IP of HTI, foreign R&D plays an almost equal role as local R&D. In terms of the impact on technological IP of HTI, foreign R&D input plays a positive role. It has a spatial spillover effect, the degree of impact is lower than that of domestic R&D input. Local governments should formulate relevant policies to encourage the fluidity of technical knowledge and overcome the sticky problem of foreign R&D technical knowledge, which is an essential aspect of absorbing foreign R&D technical knowledge in the future.

## 1. Introduction

According to the European Innovation Scoreboard 2022 released by the European Commission, compared with China, the EU’s lead in IP has narrowed. In 2022, China utilized US $189.13 billion of foreign investment, of which the high-tech industry (HTI) accounted for more than one-third. HTI is an essential field of scientific and technological competition among countries, and is based on technical knowledge or knowledge assets. However, its development requires high R&D expenditure. Backward countries that want to develop HTI face limited capital, so they need to make good use of global innovation resources. It isn’t easy for emerging economies to access cutting-edge technology through market transactions. Therefore, it is the best method to attract foreign R&D to develop China’s HTI, which is the best way to obtain global innovation resources with limited funds. The technological decomposition and the reduction of external transaction costs caused by the increase in global R&D resources make open innovation more attractive and practical in China’s manufacturing innovation. It is mentioned in the 14th Five-year Plan that enterprises are encouraged to increase investment in R&D, while relying on China’s advantages in the big market and based on the domestic cycle, attracting global innovation resources by opening up at a high level. Attracting global innovation resources from foreign R&D is an effective practice for the open innovation strategy.

As a high-quality foreign investment, foreign R&D is an essential bridge to effectively link the national innovation system with the global innovation network. The impact of foreign R&D on China’s economy and science and technology is directly related to industrial development [1]. Countries compete to attract foreign R&D, mainly focusing on the "spillover effect" brought by foreign R&D. Existing studies mainly focus on exploring the "spillover effect" of foreign R&D, and divide the "spillover effect" into symmetric spillover and asymmetric spillover. Symmetric spillover means that all enterprises in the industry have the same degree of technology leakage [2]. That is, the degree of incoming spillovers and outgoing spillovers is equal, while asymmetric spillovers refer to the degree of technology leakage being different [3]. Due to differences in absorptive capacity, geographical positioning, product differentiation, or other factors, asymmetric spillovers dominate the literature [4]. Foreign R&D is an essential part of China’s national innovation system, and an integral channel for the opening of the national innovation system [5]. Overall, the existing literature is limited to the theoretical level to verify the symmetric and asymmetric spillover effects of foreign capital. Existing literature focuses more on the relationship between local R&D and innovation output [6]. Much literature has focused on foreign direct investment (FDI) and regional or industrial innovation in China to measure the spillover effect of FDI [7], but not on foreign R&D and the industrial innovation efforts of host countries directly.

The main innovations and contributions of this paper are as follows. Firstly, past research FDI spillover effect of literature centered on FDI of China’s capacity for independent innovation, province al innovation capability, industry IP, the influence of the macro and the general research Angle, the depth of the lack of its internal mechanism, this paper further analyzes the foreign direct investment in R&D is how to impact on IP, It enriches the research perspective of FDI spillover effect. Secondly, to fully understand the new economic geography emphasizes the geographical distance and location factors on an essential role in the innovation activities, based on a variety of spatial weight matrix in measure, at the same time incorporating both domestic and foreign R&D into the unified system innovation to research, this paper enriches the research and development in the space relevance perspective the impact on IP and the existing study also relatively deficient.

HTI plays a crucial strategic role in China’s economic development [8]. HTI in developing countries under the open economic system has become an inevitable requirement for countries to enhance their national strength and competitive advantage [9]. Foreign direct investment [8], and technological innovation [8, 9] have become an essential pillar and direct driving force for the development of HTI. From the perspective of China’s HTI, this paper explores the path of foreign R&D space spillover, and studies how to induce and guide the innovation diffusion mechanism. Emerging economies should make full use of the world’s innovation resources, but at the same time maintain the principle of independent scientific and technological innovation "independent but not closed and open but not out of control". The acceleration of globalization and the evolution of the global innovation system has driven the transformation of the innovation model to a more open model. As the catalyst for the industrial development of the host country, transnational corporations provide the initial driving force for local industrialization development, and foreign companies bring advanced technical knowledge to host countries [10]. With the help of the open innovation mode, how to realize the "curve overtaking" of China’s industrial sector, especially HTI, how to realize the acceleration of technological catch-up, how to accelerate the breakthrough of advanced countries in the high-tech field blockade, and how to recognize the use of technological innovation to get rid of the industrial squeeze of backward countries, are the hot scientific research issues that need to be solved urgently.

## 2. Literature review

The literature review in this paper is mainly based on the following aspects: the measurement of IP; on the relationship between R&D and innovation.

### 2.1 The measurement of IP

Performance is often used to measure output, such as economic performance, enterprise performance, and IP. There is some literature exploring enterprise performance, which is generally measured by total factor productivity [11]. In contrast, economic performance is measured by the number of patent applications and HTI’s product exports [12]. Innovation is particularly significant for national development, so there is more and more literature on IP in recent years, and the proportion of articles on IP in emerging economies is relatively high. Literature on IP can be generally divided into enterprise IP, industrial IP, regional IP, and other perspectives, and the measurement criteria can be generally divided into two categories: Measurement of macro data and collection of questionnaires. When measuring enterprise IP, Revenue from new product sales [13], per capita sales revenue of new products [14, 15], and a number of patent applications [16–18] are often used. Of course, the sales revenue of new products and the number of authorized invention patents are also used for comprehensive measurement [19]. In general, it is consistent with the measurement of enterprise IP. When measuring industrial IP, the number of patent applications [20], the proportion of new product sales revenue [21], and other single indicators are often used to measure. There are also two indicators, the number of patent applications and the sales revenue of new products [7, 22].

### 2.2 R&D and innovation

Foreign R&D network has become a core part of the high-quality development of China’s national innovation ecology [23]. Compared with European TNCs that rely on emerging economies for offshore production, TNCs based in the United States are more willing to make knowlege-intensive foreign direct investments in emerging countries such as China and once [24]. The new growth theory believes that the driving force of economic growth comes from imitation and innovation, highlighting the critical role of factors such as R&D and externalities in innovation and high-quality economic development [25, 26]. When reviewing the literature on the relationship between local R&D and IP, the results have positive effects [27] and no effects [28]. In the past three years, the research literature has focused on the relationship between FDI and industrial innovation. It is believed that FDI significantly promotes industrial IP [7], and enterprise IP [13]. Literature studies on foreign R&D activities in Belgium found that spillover of foreign R&D usually occurs more frequently than FDI [10]. Studies have shown that foreign R&D promotes industrial innovation ability [29].

It is worth noting that TNCs enter host countries through foreign direct investment, and TNCs tend to be more productive, invest more in R&D, and generate more knowledge [30]. The innovation process relies on the province al platform caused by it. The inflow of foreign R&D stimulates the internal vitality of the province forum, and the more active its innovation is. Since technical knowledge is inherently indivisible, the successful production of new knowledge depends on acquiring external knowledge [31]. The proximity of space makes it easy to access the local knowledge base. The regional knowledge flow is closely related to tacit knowledge, which opens the process of province interactive learning. Multinational companies usually locate their innovation activities in the cluster environment, which promote the employment of professional labor [32]. Based on the HTI, from the space Angle of view, to study the foreign R&D’s spillover impact on industry IP, to learn how to make full use of the world’s innovation resources, to research how to induce and guide the diffusion of innovation, to explore how to use the open innovation model for China’s industrial sector, which especially for high technology industries "overtaking corner", those are more valuable research questions, and have the necessary theoretical implications.

**Fig 1:**
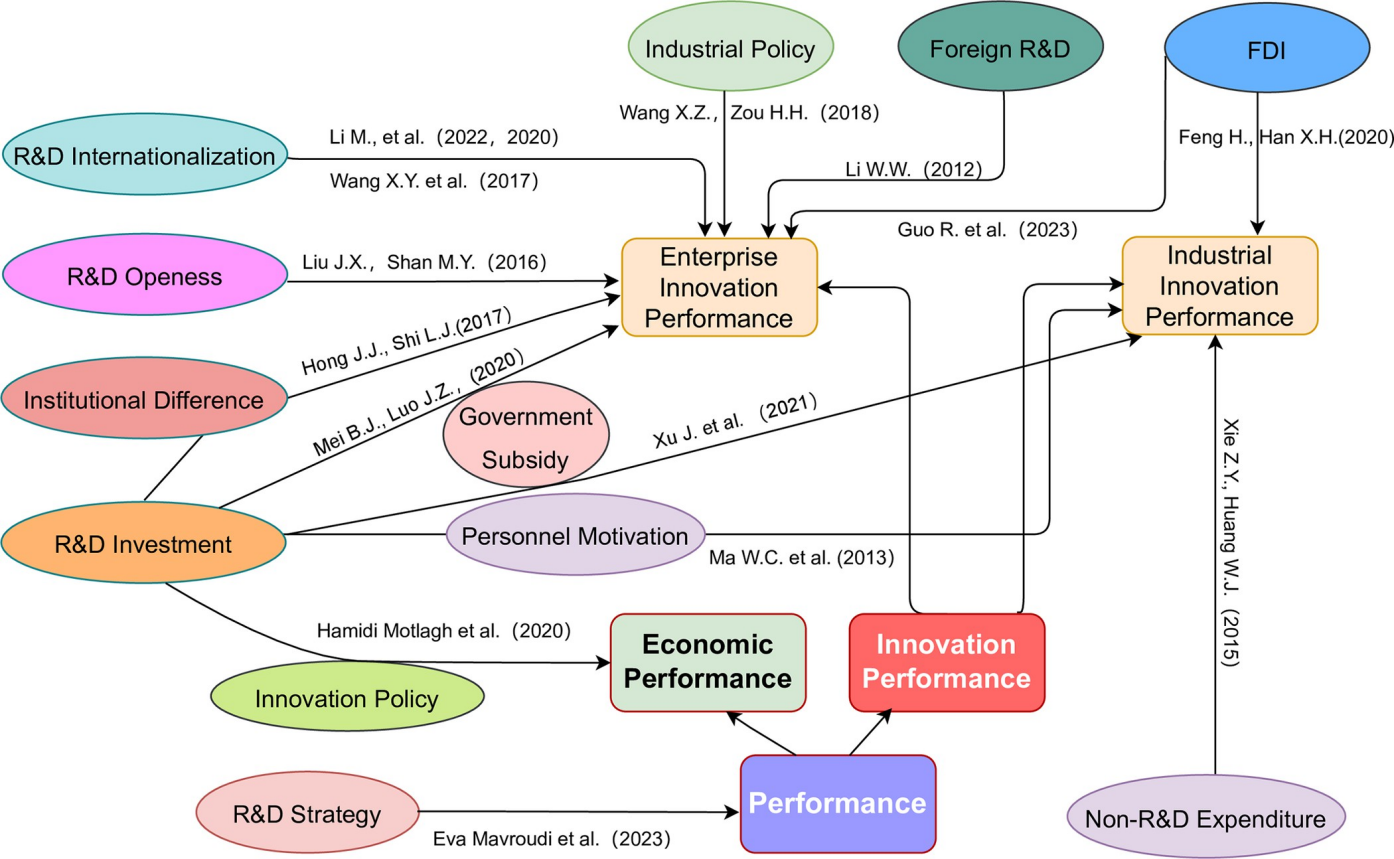
Literature collation of R&D input and IP.

This paper finds that the overall deficiencies of the existing literature are: First, although more domestic and foreign literature focused on R&D and IP, but most of the surrounding local R&D perspective to study, although also a tiny part of the literature attempts to the impact of foreign R&D perspective, the lack of appropriate research methods, spatial econometric analysis method, the reason is that although the company is bringing innovation to the market and turning it into the value of the micro main body, But even strong multinational enterprises still need to be rooted in the support and maintain its geographical location, the geographical area, support the development of the industry-related infrastructure construction, has rich experience and professional skills of human resources, more powerful knowledge base to facilitate knowledge exchange, the knowledge base constitutes the vital department of local knowledge, promoting the knowledge overflow. Therefore, we cannot study the impact of foreign R&D on IP from distance, geographical location, and location factors. Exploring the impact of foreign R&D on IP, existing research has been divided into domestic and foreign R&D. The literature only studied the impact of foreign R&D, but ignored the impact of domestic R&D. This is because the resources that produce innovative results are often not limited to the company or industry. We need to explore the improvement of IP under the open innovation mode to be closer to reality. See Fig 1 for a simple review of the literature.

## 3. Theoretical analysis

The research and development of diffusion theory can be divided into the following three branches: one focuses on the uncertainty of new technology quality [33]; the other focuses on strategic issues surrounding technology adoption [34]; the third emphasizes spillover effects and the role of learning in generating knowledge diffusion [35]. New economic geography and endogenous growth theory have typical problems: how to create new economic activities through technological innovation, and how to choose the location of these activities [36]. Localized technical knowledge emphasizes the role of a joint product of economic and production activities. It highlights the role of external knowledge as the primary input of general new knowledge production. Because of the inherent inseparability of technical knowledge, the successful production of new knowledge depends on acquiring external knowledge. Spillover effects may encourage R&D spending under certain conditions, and R&D also becomes more critical to absorb spillover knowledge. Spillovers are spatially limited [37]. In other words, although some technologies can be codified, a large amount of technical knowledge is "implicit," so knowledge spillover is geographically bounded, and the spillover of "implicit" knowledge needs to be close. The knowledge brought by foreign R&D has spillover impact on the IP of HTI through demonstration effect, talent flow effect, and competition effect.

First, the localized technical knowledge produced by foreign investment in R&D provides demonstration effects for the host country’s industry. Research and development are considered the main driving force of innovation. Due to the double-sided nature of foreign investment in R&D, it is the main driving force of innovation of multinational companies and the interface with the local external environment. Global companies have more productivity, invest more in research and development, and generate more technological know-how. After acquiring superior knowledge and more advanced technology, global companies embed their R&D in local areas, injecting vitality into local industry development and generating localized technical knowledge through R&D, which connects with the local external environment. On the one hand, with the help of the demonstration effect, TNCs provide positive externalities of technical knowledge for developing local industries in host countries. Therefore, through the demonstration effect, TNCs make the knowledge generated by foreign R&D spill over to the local province, and provide "tacit" knowledge for imitation and learning for the local HTI. Demonstration effects also shorten the trial-and-error process of finding inventions for local industries. On the other hand, the neighboring provinces are led by the leading areas. They actively imitate their development models and learn their innovative knowledge, leading to the indirect knowledge spillover of foreign R&D. Therefore, the demonstration effect of foreign R&D spills over to the local and adjacent areas, which has a spillover impact on improving the host country’s industrial IP.

Second, the talent cultivated by foreign R&D provides the talent flow effect for the host country’s industry flow. Foreign R&D has promoted the employment of a professional labor force, trained innovative talents with professional technical knowledge, and helped the host country improve the innovation skills of the labor force. China continues to expand its opening to the outside world and to attract multinational companies into a large domestic market scale. TNCs with advanced technology and management experience need to mass production in the host country, to conform to the local consumer demand or a global company, to update the advanced equipment for subsidiaries, and to adopt new technologies, such as requirements. That is bound to a large number of research and development in host countries. This process is accompanied by the hiring of labor in the host country. On the one hand, the R&D personnel in global companies have caused the talent flow effect in the host country through R&D cooperation, exchange, labor migration, and other channels. The technological knowledge that is invisibly attached to the innovative talents, is brought to other enterprises in the host country through the talent flow effect. That brings a lot of new knowledge for improving industrial IP. On the other hand, if the talent flow effect mentioned above is obtained to neighboring provinces, or if the talents from neighboring provinces enter the area through the flow and start the interactive learning process through specific channels. The spatial diffusion barrier of knowledge spillover from multinational corporations will be reduced, and the IP of industries in neighboring provinces of the host country will be improved. Talent flow also has the characteristics of geographic distance. Of course, the multinational companies, to reduce the knowledge overflow, will pay higher wages to prevent brain drain, but on the whole, foreign R&D talent flow effect produced by the embedded process for the host country of science and technology talent, has brought the spillover effect, increase the industry IP of the host country.

Third, FDI promotes the host country’s industrial IP through the competition effect. The host country uses the technology market, and enterprises gain sustainable competitive advantages through pressure and challenges. Positive pressure from competitors can stimulate innovation, which is the fear of lagging and the desire for the leading position of enterprises. On the one hand, the competitive effect generated by the embedding of foreign R&D makes the technological knowledge spread well among the competitors. It provides a place for local enterprises in the embedding province of the host country to "learn in parallel" from transnational corporations. The competition will improve IP. On the other hand, the neighboring areas effect by learning from the spillover knowledge absorbed by the local area. Of course, with the increasing amount of externality knowledge available within the province, competition is positive for improving IP. The cost of knowledge governance due to the congestion effect of coordinated provinces will increase, with the increase in the number of enterprises accessing the same knowledge pool. That is, the entry of multinational corporations will also cause negative externalities related to competition, namely the "market stealing effect." The market generates congestion costs, resulting in income inequality and low growth.

In this study, we start from the diffusion theory, combined with establishing an open regional innovation ecosystem, and explore the open innovation mode; foreign R&D will effectively connect the regional innovation ecosystem with the global innovation network, and brings available technical knowledge to the localized knowledge pool. Foreign R&D promotes the local industry IP process. Unlike previous literature on FDI spillover effects, this paper focuses on the spillover effects of foreign research and development, because spillover effects of foreign R&D occur more frequently than those of FDI [10]. At the same time, compared with the previous studies on the interaction between competition and cooperation between foreign companies and domestic companies, which resulted in the innovation output of local companies being different [38]. This paper mainly explores the knowledge brought by local R&D activities of foreign companies to local industrial innovation, and the research perspective is also different from the theoretical level. In addition, this paper explores the spatial spillover effect of foreign R&D, enriching the practical application of diffusion theory and injecting fresh blood into the research field of spillover effect.

## 4 Research design

### 4.1 Model establishment

This paper adds spatial econometric analysis. Of course, standard spatial econometric models include the spatial Dubin model, spatial lag model, and spatial error model. The specific formulas are set as follows:

$$lnY_{it}=\rho \sum_{j=1}^{n}W_{ij}lnY_{it}+\lambda_{1}lnX_{1}+\lambda_{11}\sum_{j=1}^{n}W_{ij}lnX_{1}+\gamma_{2}{lnX}_{11}{+\gamma }_{22}\sum_{j=1}^{n}W_{ij}lnX_{11}+C+\varepsilon$$ (1)

$$lnY_{it}=\rho \sum_{j=1}^{n}W_{ij}lnY_{it}+\lambda_{1}lnX_{1}+\gamma_{2}{lnX}_{11}+C+\varepsilon ,\;\varepsilon ∼(0,\;\sigma^{2}In)$$ (2)

$$lnY_{it}=\lambda_{1}lnX_{1}+\gamma_{2}{lnX}_{11}+\varepsilon ,\;\varepsilon =\lambda W\varepsilon^{’}+\mu$$ (3)

In the above formula, Y represents the explained variable, named IP of China’s HTI. X1 represents foreign R&D. X’ represent other variables, including domestic R&D, workers in China’s HTI, the HTI export delivery value, technical support of the local government, province social capital, and HTI technical reconstruction spending, six indicators. C is the constant, andεis the regression residuals. ρ, λ, andγare coefficient. W is the weighting matrix for the space.

The selection criteria of the above three spatial measurement models are as follows: LM test, Hausmann test, and LR test are required when the Moreland index is significant. The most appropriate spatial measurement model is selected according to the conclusions of the above three tests, and the spatial spillover effects of the corresponding explained variables are tested. The selection criterion of the specific model is the LM test: it is necessary to check whether the LM error and LM lag statistics pass the test. If the LM-lag test is significant, the SAR model is selected; if LM-error test is significant, the SEM model is selected; if both pass significantly, robust LM-lag and LM-error test are performed; if robust the LM-lag test is significant, the SAR model is chosen; if robust LM-error test is significant, the SEM is chosen. The SEM model is chosen if all are significant. If neither test passes, the OLS model is chosen. Hausmann test: after the LM test, it is necessary to determine whether the chosen spatial measurement model should be a fixed effect or random effect. If fixed effects are identified, it is necessary to further determine whether to choose mixed fixed effects, individual fixed effects, or time fixed effects. It is worth noting that if the SDM model is selected, the LR test should be carried out to determine whether the SDM model will degenerate into the SAR or SEM model.

### 4.2 Spatial weight matrix

The spatial weight matrix W is the crucial premise of spatial measurement. It is composed of spatial data of N provinces. The specific form of the spatial weight matrix is as follows:

$$W=\left(\begin{array}{ccc} W_{11} & \cdots & W_{1n}\\ \vdots & \cdots & \vdots \\ W_{n1} & \cdots & W_{nm} \end{array}\right)$$

Among them, Wij represents the distance, and those on the main diagonal represent the distance to the same province, both marked as 0. The spatial weight matrix reflects the spatial distance between areas. There are various types of spatial weight matrices used in spatial econometric analysis. This paper mainly studies the spatial spillover effect of technological knowledge brought by foreign R&D on the industrial IP of the host country. To avoid the unicity of the adjacency matrix, which is based on the adjacency relationship, and to ensure more accurate empirical results of spatial econometric model evaluation, this paper constructs distance matrix W2 which based on distance, and nested matrix W3 which based on geographical and economic distance. This paper selects three W for spatial econometric analysis. W1 space adjacency matrix is based on Queen, namely adjacency matrix; when province I borders province J, Wij marks as 1, otherwise as 0. W2 is the distance matrix, which Wij is calculated using the reciprocal square of road mileage between provincial capitals of province I and J. W3 is a nested matrix, and its Wij is composed of the matrix converted by the proportion of the average GDP of each province in the average GDP of the whole country, multiplied by the product of the diagonal matrix transformed by the longitude and latitude of the province I and J. The nested matrix based on geographical and economic distance is generated using the longitude and latitude of 23 provinces and the ratio of the average GDP of each province during the sample period. The province and adjacent provinces. W3 is used for robustness testing.

### 4.3 Variable selection

#### 4.3.1 Explained variables

As an explained variable, industrial IP refers to IP of the HTI. The measurement methods of industrial IP are not uniform, and some scholars use patent output or new product sales revenue as a single index to calculate it. Some scholars also use the proportion of new product sales output value to calculate, and some scholars use industrial output value profit and tax rate, industrial input and output indicators to measure the total expenditure of industrial science and technology activities. Some scholars even use the number of patents and sales of new products for comprehensive evaluation. This paper refers to the comprehensive measurement index [7], which considers an application for a patent for an invention for HTI (technology innovation), HTI’s new product sales income (product innovation), and HTI’s effective number of invention patents (technology innovation 1). In this paper, the number of effective invention patents in the HTI is selected for the robustness test.

#### 4.3.2 Explanatory variables

As a core explanatory variable, scholars at home and abroad have different measurement methods for further research standpoints in measuring this index. Some start from the perspective of foreign R&D capital embedding and use the proportion of foreign R&D capital stock to measure it. The ratio of foreign R&D expenditure is also used to measure the intensity of foreign R&D. This paper adopts the internal expenditure of R&D funds of foreign-funded enterprises in HTI as the measurement index [39]. It adopts the R&D of foreign-funded enterprises in different provinces of China as the measurement index. This index is measured by the sum of the internal R&D expenditure of foreign-invested enterprises in China Hong Kong, Macao and Taiwan, and the unit is ten thousand yuan. In particular, since the Statistical Yearbook of China’s HTI 2018 has not been published and due to statistical standards, there is no data on the internal expenditure of foreign R&D funds by province before 2007. Therefore, the data are mainly from the statistical data by the province in the Statistical Yearbook of the HTI from 2008 to 2017. Thus, the study only covers the panel data of the HTI from 2007 to 2016, and all other variables are selected in this time range.

#### 4.3.3 Control variables

The control variable adopts six indexes: local R&D, HTI’s employees, HTI’s export trade, local government science and technology support, province social capital, and HTI’s technological transformation expenditure.

The local R&D is calculated by the R&D of domestic enterprises in HTI. The HTI employees are measured by the annual average of the HTI’s employees. The government plays the role of middleman, the connection of demand, and the promotion of innovation in the formulation and promotion of reliable innovation policies. Therefore, government science and technology support adopts the measurement method of most scholars and adopts the local financial expenditure on science and technology (ten thousand yuan) to measure. Payment on technological transformation of the HTI mainly refers to the spending on backward technology and equipment mentioned by advanced technology and equipment of the HTI. Therefore, it is measured by the expense incurred by technological transformation during the reporting period of the HTI, and the unit is ten thousand yuan. The export trade of HTI is measured by the export delivery value of the HTI.

Province social capital: Social capital is the prerequisite for innovation, knowledge creation, and transfer of conditions. It stimulates human interaction and the relationship between network flow. The formation of the valuable knowledge innovation is embedded in the process of the accumulation of social backgrounds. The measured method of social capital is affected by data availability, which is no agreement in academia. This paper uses the internet penetration rate and Internet users per million people to measure [40] the internet broadband access port (ten thousand).

### 4.4 Data sources

The 2007–2016 data on local R&D, foreign R&D, number of employees in the 23 provinces of the HTI, export delivery value of HTI, and technological transformation expenditure of the HTI are all from the statistical Yearbook of China’s HTI from 2008 to 2017. The data on the 23 provinces’ manufacturing employees come from China’s Labor Statistics Yearbook, while the data on local financial expenditure on science and technology and province social capital come from the provincial statistical yearbook. To avoid the endogeneity of variables, the above variables are logarithmic.

In particular, Jilin province, Hainan province, Xinjiang uygur autonomous province, Ningxia hui autonomous province, Qinghai province, Tibet autonomous province, Inner Mongolia autonomous province, Gansu province are for many years without the inflow of foreign R&D data, combined with mainly study the influence of the foreign research and development of industrial IP, so the above regions are excluded in this paper. Meanwhile, since the Statistical Yearbook of China’s HTI in 2018 has not been published, to ensure the consistency of research data, the overall variables only cover the panel data of the HTI in 23 provincial provinces from 2007 to 2016. The data are presented in Table 1.

**Table 1:** Descriptive statistics of each variable.

variable	the mean	the standard deviation	the minimum value	the maximum	number of observations
**technology innovation**	7.0644	1.4766	4.1744	10.8477	230
**technology innovation 1**	6.8362	1.5598	3.5835	11.9350	230
**product innovation**	14.9385	1.6598	11.3858	18.8026	230
**foreign R&D**	10.4924	2.2606	2.9957	14.4255	230
**local R&D**	11.9457	1.3826	7.3746	15.7160	230
**industrial worker**	3.2386	1.0614	0.9226	5.9647	230
**industrial export trade**	15.0821	2.0720	11.0349	18.9708	230
**local financial support for science and technology**	13.2721	0.9099	11.3783	15.821	230
**local social capital**	6.7686	0.8789	4.5695	8.7820	230
**funds for industrial technological transformation**	10.9254	1.3578	6.6995	13.8930	230

## 5. Empirical analysis

### 5.1 Full-sample test results

Overall, the panel data of 23 provincial provinces in China from 2007 to 2016 are analyzed using the spatial econometric analysis method and Stata 15.1, except for the eight missing provinces of Jilin, Hainan, Xinjiang, Ningxia, Qinghai, Tibet, Inner Mongolia, and Gansu.

#### 5.1.1 Spatial autocorrelation

The spatial weight matrices are W1 and W2, respectively. Product innovation and technological innovation are used to measure IP of the HTI, and the global Moran index of IP of the HTI is calculated, indicating the existence of spatial autocorrelation, which can be used for spatial econometric analysis. The Moran index of the variables is shown in Table 2.

**Table 2:** Global Moran’s I of IP.

year			2007	2008	2009	2010	2011	2012	2013	2014	2015	2016
**product innovation**	W_1_	Moran’s I	0.196**	0.172**	0.155*	0.194**	0.214**	0.214**	0.195**	0.160*	0.166**	0.182**
		Z test	1.844	1.666	1.537	1.855	2.015	2.019	1.864	1.597	1.647	1.789
	W_2_	Moran’s I	0.174**	0.121*	0.121*	0.125*	0.139*	0.156**	0.135*	0.101	0.089	0.104
		Z test	1.808	1.378	1.378	1.424	1.546	1.687	1.509	1.230	1.129	1.264
**technology innovation**	W_1_	Moran’s I	0.093	0.022	0.085	0.127*	0.169**	0.083	0.130*	0.123*	0.111	0.135*
		Z test	0.385	0.188	1.054	1.367	1.697	1.013	1.388	1.341	1.245	1.434
	W_2_	Moran’s I	0.023	0.026	0.079	0.112*	0.150**	0.111*	0.124*	0.098	0.093	0.095
		Z test	0.194	0.613	1.080	1.340	1.663	1.323	1.445	1.235	1.186	1.206

Note

*,**,***represent significance at 10%, 5%, and 1% levels, respectively(the following tables are also marked in this way); The Moran index of technological innovation 1 was significantly positive in 2010 and 2010, which is not listed in the above table.

#### 5.1.2 Empirical analysis of spatial spillover effect

Before the spatial spillover effect analysis, the LM test found that the LM results of the variable, which measures technological innovation, were shown in Table 3. The LM Error statistic passed the 1% level test under two different spatial weight matrices, while the LM Lag statistic did not pass the test, the SEM model was selected. In measuring the product innovation, the LM Error and LM Lag statistics did not pass the test under two different spatial weight matrices when measuring the sales revenue of new products, the OLS model was selected. When measuring product innovation by new product development expenditure, W1 used as the spatial weight matrix, LM error and LM Lag statistics, Robust LM error and Robust LM Lag statistics all passed the test at 1% level, the SDM model was selected as the optimal model, and when tested again by the LR whether the SDM could degenerate into the SAR and SEM. It was found that the SDM was rejected to degenerate into the SEM, so the SEM model was finally selected. At the same time, product innovation is measured by new product development expenditure, W2 as a spatial weight matrix, the LM error single statistic passes the test at 1% level, and the SEM model is selected. The LM test results of the model are shown in Table 3.

**Table 3:** LM test results of each variable.

statistic		the inspection results (W1 / W2)	p values
**technology innovation**	LM error test	7.443***/ 7.059***	0.006/0.008
	LM lag test	0.461/0.155	0.497/0.693
**technological innovation 1**	LM error test	16.171***/ 10.959***	0.000/0.001
	LM lag test	0.219/0.574	0.639/0.449
**product innovation**	LM error test	0.539/1.032	0.463/0.310
	LM lag test	0.351/0.630	0.554/0.427

Note: W was used before and after semicolons in the test results from W_1_ and W_2_ space weight matrix calculation.

Next, the Hausmann test is carried out. According to the test results in Table 4, the Hausmann-Test results of all measurement indicators of IP are optimized by adopting a fixed effect. Furthermore, The LR test is carried out to compare the most significant among mixed fixed effect, individual fixed effect, and time fixed effect. By comparing the results of the double fixation effect and time fixed effect, the overall choice of individual fixation is the best. Other test results are shown in Table 4.

**Table 4:** Hausman test and LR test results.

statistic		the inspection results	p values
**technology innovation**	Hausman test	21.69	0.0029
	LR test (lrtest both ind)	9.56/12.41	0.4796/0.2584
	LR test (lrtest both time)	163.39/161.91***	0.0000/0.0000
**technological Innovation 1**	Hausman test	16.15	0.0238
	LR test (lrtest both ind)	17.26/6.40	0.0687/0.7804
	LR test (lrtest both time)	211.43/212.95***	0.0000/0.0000
**product innovation**	Hausman test	18.27	0.0108
	LR test (lrtest both ind)	24.61***/ 23.48**	0.0061/0.0091
	LR test (lrtest both time)	145.71***/ 113.73***	0.0000/0.0000

Note: W was used before and after the semicolon of the test results from W_1_ and W_2_ to calculate.

After the LM test, the Hausmann test and LR test, the SEM model, and individual fixed effect are the best variables for measuring technological innovation. The OLS model was selected when new product sales revenue was used to measure product innovation. In contrast, the SEM model and individual fixed effect were selected when new product development expenditure was used to measure product innovation 1. Then, the spatial effect was analyzed, and the results are shown in Table 5.

**Table 5:** Influence and spatial effect of foreign R&D on IP of HTI.

explained variable	innovation performance (technology innovation)					innovation performance (product innovation)
	OLS	SEM-W_1_	SEM-W_2_	SAR-W_1_	SAR-W_2_	OLS
**foreign R&D *investment***	0.0700 **	0.0828 ***	0.0794 ***	0.0755 **	0.0749 **	0.1460 ***
	(2.13)	(2.74)	(2.71)	(2.47)	(2.49)	(3.83)
**local R&D *investment***	0.2014 ***	0.1885 ***	0.2002 ***	0.1752 **	0.1631 **	0.1533 *
	(2.72)	(2.82)	(3.05)	(2.51)	(2.37)	(1.79)
**industrial worker**	0.2681 *	0.2509 *	0.1721	0.2538 *	0.2146	0.6852 ***
	(1.70)	(1.72)	(1.18)	(1.74)	(1.48)	(3.76)
**industrial export *trade***	-0.1460 ***	-0.3140 ***	-0.1228 **	-0.1389 ***	-0.1328 ***	-0.0842
	(-2.77)	(-2.63)	(-2.52)	(-2.84)	(-2.75)	(-1.38)
**local financial support for science and technology**	0.4683 ***	0.3987 ***	0.3828 ***	0.4111 ***	0.3875 ***	0.0207
	(3.78)	(3.37)	(3.24)	(3.46)	(3.33)	(0.14)
**local social capital**	0.2076 **	0.2502 ***	0.2700 ***	0.1473	0.1123	0.1771
	(2.14)	(2.60)	(2.85)	(1.54)	(1.18)	(1.58)
**funds for industrial technological transformation**	0.0753 *	0.0813 **	0.0750 **	0.0808 **	0.0767 **	0.0802 *
	(1.94)	(2.31)	(2.15)	(2.25)	(2.17)	(1.79)
**lambda or rho**		0.2175 **	0.3004 ***	0.1510 *	0.2402 ***	
		(2.22)	(3.04)	(1.79)	(2.82)	
**sigma2_e**		0.0991 ***	0.0967 ***	0.1003 ***	0.0977 ***	
**R** ^**2**^	0.8303	0.8270	0.8119	0.8156	0.7965	0.8645
**fixed effect**	Yes	**individual fixed**				Yes
**sample size**	230	230	230	230	230	230

Note: Z values are in brackets(the following tables are also marked this way); The following table is the same. The OLS constant terms are not included.

According to the results of spatial effect, foreign R&D in HTI has a significant positive spatial spillover impact on IP of the HTI. When IP of HTI is measured by technological innovation, HTI’s IP is affected by the positive spatial spillover effect of adjacent geographical provinces. Compared with the research that R&D input of multinational corporations positively promotes China’s industrial innovation capability [29], this study puts forward that there is still a spatial spillover effect of foreign R&D. In other words, local foreign R&D has a positive impact on the regional industrial IP. Meanwhile, neighboring regions, led by leading areas, actively imitate their development mode and learn their innovative knowledge, resulting in indirect knowledge spillover of foreign R&D to neighboring areas. In conclusion, the demonstration effect of foreign R&D spills over to the local and neighboring regions, resulting in a spillover effect for improving industrial IP in the host country. When IP of HTI is measured by product innovation, there is no spatial spillover effect, which also verifies that knowledge in the innovation system is written and mature to a large extent. It mainly develops and flows along the track oriented by technological innovation. However, foreign R&D has a positive impact on IP of HTI, and it is significant at the 1% level. Therefore, open innovation has a significant to a certain extent. There is a certain spatial spillover effect. Compared with foreign R&D, domestic R&D has a more significant positive impact on IP of HTI, which is also the purpose of the state to encourage the cultivation of independent innovation ability.

The effect of HTI employees on IP of HTI is generally positive at 10%. Employees have a positive promoting impact on the industrial IP of the local region. The talent flow effect, which brings technical knowledge to the neighboring region, or starts the interactive learning process through certain channels after talents from the neighboring region flow into the local region, reduces the spatial diffusion barrier of knowledge spillover from transnational corporations of the host industry. Improve the industrial IP in the neighboring regions of the host country. On the whole, the talent flow effect generated by the embedding process of foreign R&D has cultivated scientific and technological talents for the local and neighboring regions of the host country, brought about a spillover effect, and improved the industrial IP of the host country.

It is worth noting that the local financial expenditure on science and technology significantly promotes IP of HTI at 1%. This factor has a high impact on IP of HTI. Adopting advanced technology and equipment in HTI has a significant positive impact on IP of HTI, and province al social capital also plays a positive role in upgrading IP of HTI. However, the export behavior of HTI plays a significant role in upgrading IP of HTI. This also validates the effect of exports on IP, with two types of facilitation and inhibition [41], and perhaps more facilitation in advanced economies. Compared with developed economies, the export volume of emerging economies is relatively tiny. With fierce international market competition, export inhibits industrial IP [42]. Therefore, the development of China’s HTI needs to launch innovations before international competitors reduce existing products or services to mass commercialization.

### 5.2 Further analysis

To further analyze the influence of foreign R&D on IP of HTI in the 23 provinces, and the overall IP of HTI in the areas, the SAR model empirical results of different spatial weight matrix analyses are decomposed into direct effect, indirect effect, and total effect, which as shown in Table 6. The foreign R&D has a significant positive promoting impact on IP of HTI in this area, and also has a positive, stimulating impact on the spillover effect of IP of HTI in neighboring areas. It is not obvious at present, but has a significant positive promoting impact on the overall IP of HTI in this province. Therefore, at present, the influence of foreign R&D on IP of HTI is mainly restricted by the region, which has a significant impact on this region, which also proves that R&D institutions in neighboring areas need to jointly locate with foreign R&D institutions in leading areas (Hansen Teis and Hansen Ulrich Elmer, 2020). Or through joint research and development, neighboring regions can better absorb the advanced knowledge brought by foreign research and development in leading regions. For example, Beijing attracts a lot of foreign R&D, and foreign R&D brings advanced technical knowledge to the area. To obtain more advanced technical knowledge, local R&D institutions in Tianjin or Hebei, which are adjacent to Beijing, can set up their R&D institutions near the foreign R&D institutions in Beijing, to gain better access to the knowledge spillover brought by foreign R&D.

**Table 6:** Decomposition of direct effect, indirect effect and the total effect of the SAR model.

Innovation performance		The direct effect	The indirect effect	The total effect
W_1_	**Foreign R&D**	0.0771 **	0.0136	0.0907 **
		(2.44)	(1.27)	(2.36)
	**Local R&D**	0.1734 **	0.0287	0.2021 **
		(2.56)	(1.38)	(2.59)
W_2_	**Foreign R&D**	0.0771 **	0.0234	0.1005 **
		(2.46)	(1.60)	(2.38)
	**Local R&D**	0.1624 **	0.0477 *	0.2101 **
		(2.42)	(1.71)	(2.43)

We note that, the R&D of domestic enterprises has a significant positive impact on IP of HTI in the local province, and the spillover impact on IP of HTI in the neighboring province is occurring, although it is only significant at the level of 10%. On the whole, it is necessary to explore the open independent innovation mode under the premise that the innovation of HTI in China is mainly encouraged by independent innovation. Of course, technical knowledge is sticky in the area where it is produced. Technical knowledge is stickier than production, but it also has leakiness and mobility. Joint site selection, joint research and development, and the establishment of a R&D strategic alliance are all measures to overcome the stickiness of technical knowledge. These measures often increase the fluidity of technical knowledge and make it more spatial spillover effect. The results of effect decomposition are shown in Table 6.

### 5.3 Robustness test

To verify the reliability of the results, the robustness tests were performed by substituting explained variables and spatial weight matrices. The first step is to replace the explained variables with valid invention patents in HTI for the robustness tests. In this paper, technological innovation 1 is used to replace the explained variable technological innovation. The results are shown in Table 7. The coefficients, directions, and significance of the core explanatory variables are generally consistent, which verifies the stability of the results.

**Table 7:** The spatial effect of foreign R&D on IP of HTI.

explained variable	replace the explained variable				replace the explained variable and spatial weight matrix
	SEM-W_1_	SEM-W_2_	SAR-W_1_	SAR-W_2_	SAR-W_3_
**foreign R&D**	0.0503 *	0.0487 *	0.0572 **	0.0536 **	0.0564 **
	(1.88)	(1.84)	(2.21)	(2.10)	(2.16)
**local R&D**	0.2884 ***	0.2884 ***	0.2230 ***	0.2098 ***	0.1986 ***
	(4.68)	(4.67)	(3.63)	(3.44)	(3.12)
**industrial worker**	0.0071	0.0092	0.0135	0.0100	0.0278
	(0.05)	(0.07)	(0.11)	(0.08)	(0.22)
**industrial export trade**	-0.0773 *	-0.0810 *	-0.0742 **	-0.0772 *	-0.0766 *
	(-1.73)	(-1.86)	(-1.79)	(-1.89)	(-1.84)
**local financial support for science and technology**	0.2534 **	0.2661 ***	0.1853 *	0.1979 **	0.1875 *
	(2.45)	(2.59)	(1.86)	(2.03)	(1.88)
**local social capital**	0.7646 ***	0.7568 ***	0.4713 ***	0.4193 ***	0.3374 ***
	(9.15)	(9.22)	(4.83)	(4.23)	(2.86)
**funds for industrial technological transformation**	0.0425	0.0433	0.0576 *	0.0527 *	0.0629 **
	(1.35)	(1.37)	(1.88)	(1.75)	(2.04)
lambda or rho	0.1741 *	0.2187 **	0.3246 ***	0.3754 ***	0.4297 ***
	(1.68)	(2.13)	(4.49)	(5.05)	(4.54)
sigma2_e	0.0787 ***	0.0779 ***	0.0723 ***	0.0705 ***	0.0730 ***
R^2^	0.7900	0.7900	0.7492	0.7346	0.7400
fixed effect	Individual fixed	Individual fixed	Individual fixed	Individual fixed	Individual fixed
sample size	230	230	230	230	230

In the second step, we use the W3 (nested matrix), that is, the longitude and latitude of 23 provinces, and the mean value of GDP of each province in the overall GDP of China are used to generate the nested matrix W3. W3 is based on geographical and economic distance as the spatial weight matrix. At the same time, we replaced the explained variables for the robustness test, and the specific results are shown in Table 7. The coefficient, direction, and significance of the core explanatory variables are generally consistent, which verifies the stability of the results. The robustness test results are shown in Table 7.

It is worth noting that the robustness test shows that foreign R&D has a significant positive impact on IP of HTI in the province, IP of HTI in neighboring provinces, and R&D in the whole sample. In other words, by replacing the explained variables with valid invention patents in HTI, we find that the spatial spillover effect of foreign R&D has become prominent, and it is significant at 10%. Of course, compared with the influence of foreign R&D, the positive spatial spillover effect of domestic investment on IP of neighboring HTI still plays a dominant role. However, with the inflow of open innovation resources, foreign R&D is another effective way to improve IP of China’s HTI. The effect decomposition in the robustness test is shown in Table 8.

**Table 8:** Direct effect, indirect effect, and total effect of SAR model decomposition.

Innovation performance		The direct effect	The indirect effect	The total effect
W_1_	**Foreign R&D**	0.0598 **	0.0270 *	0.0868 **
		(2.18)	(1.75)	(2.12)
	**Local R&D**	0.2263 ***	0.0990 ***	0.3254 ***
		(3.76)	(2.86)	(3.88)
W_2_	**Foreign R&D**	0.0564 **	0.0316 *	0.0881 **
		(2.08)	(1.73)	(2.02)
	**Local R&D**	0.2142 ***	0.1168 ***	0.3311 ***
		(3.58)	(2.85)	(3.68)

## 6. Conclusions and recommendations

China’s stable domestic investment environment continues to be a hot spot for attracting FDI, especially in the context of the COVID-19 epidemic in 2020; China continues to become the world’s largest recipient of foreign investment, while attracting the participation in foreign research and development centers. Based on the panel data of HTI in 23 provinces in China from 2007 to 2016, this paper explores the influence process of foreign R&D on IP of HTI in China, by using the spatial econometric analysis method, and draws the following conclusions:

(1) According to the results of spatial effect, the open innovation model formed by HTI attracting foreign R&D has a significant promotion impact on IP of HTI in China to a certain extent, and there is a particular spatial spillover effect. The FDI attracted by HTI has a positive impact on IP of local HTI, and the FDI has a significant positive spatial spillover impact on IP of HTI. IP of HTI is affected by the positive spatial spillover effect of adjacent geographical provinces. When product innovation is used to measure IP of HTI, there is no spatial spillover effect. Still, foreign R&D investment has a positive promoting impact on IP of HTI, and it is significant at 1% level.

(2) Compared with foreign R&D, domestic R&D still plays a dominant role in promoting IP of HTI. The R&D input of domestic enterprises has a positive effect on the spatial spillover effect of local HTI’s IP and neighboring HTI’s IP. On the whole, it is necessary to explore the open independent innovation mode under the premise that the innovation of HTI in China is mainly encouraged by the independent innovation, which is also the purpose of the state to promote the cultivation of independent innovation ability. Of course, the research also found that the factors such as employees, local financial expenditure on science and technology, province social capital and the technological transformation process of HTI significantly promote HTI’s IP of HTI. In contrast, the export behavior of the HTI plays a significant inhibiting role in promoting HTI’s IP of HTI.

(3) Foreign R&D has a significant positive effect on IP of HTI in this province. The spillover effect of foreign R&D on IP of HTI in neighboring provinces also has a positive promotion effect. At present, the promotion effect is not significant. Still, in the process of the robustness test, it is found that the spatial spillover effect of foreign R&D on neighboring areas has been highlighted, and it is significant at the level of 10%. Foreign R&D has a significant positive impact on IP of HTI in this province, IP of HTI in neighboring provinces and the overall IP of HTI. Although local R&D still plays a leading role in influencing the IP of HTI, foreign R&D, as the inflow of open innovation resources, plays an increasingly prominent role in promoting IP of HTI in the host country, which through the role of R&D’s spillover effect and spatial spillover mechanism. It is also another effective way to improve IP of China’s HTI.

In this paper, we use the spatial measurement method to explore the influence of foreign R&D on IP of HTI in host countries. It is found that there is a positive spatial spillover effect, that is, foreign R&D positively promotes IP of HTI in the region, and also has a spatial spillover effect on neighboring areas. Compared with previous literature, it highlights the "spatial" spillover effect of technological knowledge brought by foreign R&D. Secondly, it compares the contribution of foreign R&D and domestic R&D to IP of HTI in emerging economies, which adds new content to the research field of IP.

Of course, this paper also has some limitations. First, the data analyzed empirically in this paper is from 2007 to 2016. Due to the change in statistical caliber, the critical variables after 2017 cannot be found, so the research results have certain limitations. Second, the measurement of IP is the macro data from the statistical yearbook of China’s HTI, rather than the first-hand data obtained from a questionnaire survey. Therefore, in the empirical analysis, the measurement of explained variables is one-sided. Third, this paper only studies the problem of attracting foreign R&D to HTI in China, one of the emerging economies, and does not compare it with developed economies. Therefore, the empirical results only apply to China, and the scope of application has certain limitations.

Future research can be explored from the following aspects: First, from the perspective of evolutionary economics, we can deeply explore the interaction between local R&D and foreign R&D systems on IP of HTI. This direction can highlight that innovation is the result of the interaction between innovation subjects and the environment. Second, the influence of industrial policies or institutional quality can be included. After all, there is still a certain gap between the institutional quality of emerging economies and that of developed economies. Third, we can attempt to collect questionnaires to measure IP to overcome the lack of panel data in the latest years.
